# Muslim communities’ perspectives and preferences regarding end-of-life symptom management: a systematic review and narrative synthesis

**DOI:** 10.1136/bmjopen-2025-108877

**Published:** 2026-01-14

**Authors:** Joodi Mourhli, Krzysztof Sosnowski, Isla Kuhn, Ben Bowers

**Affiliations:** 1 Department of Public Health and Primary Care, University of Cambridge, Cambridge, UK; 2 School of Clinical Medicine, University of Cambridge, Cambridge, England, UK; 3 Medical Library, University of Cambridge, Cambridge, UK

**Keywords:** PALLIATIVE CARE, Systematic Review, Islam, Patient-Centered Care

## Abstract

**Abstract:**

**Objectives:**

To provide a synthesis of the published research evidence on Muslims’ perspectives and preferences regarding end-of-life symptom management to inform future practice and research priorities aimed at providing sensitive end-of-life care.

**Design:**

Systematic review and narrative synthesis.

**Data sources:**

MEDLINE, EMBASE, CINAHL, PsycINFO, Web of Science, ASSIA, The Cochrane Library and Global Health were searched from 1 January 1994 to 10 July 2024, alongside reference searches of included papers and hand searches of two journals.

**Eligibility criteria:**

The included papers presented primary research on end-of-life care among Muslims in the British Isles.

**Data extraction and synthesis:**

Data were collected on publication details, study aims, participants, methods and results. Studies were appraised using Gough’s weight of evidence framework. An inductive narrative synthesis consisting of three steps was conducted. This involved conducting a preliminary synthesis of findings, exploring relationships between studies and assessing the robustness of the synthesis.

**Results:**

18 papers were included in the synthesis. Patients prioritised conformity between religion, culture and end-of-life symptom management. Symptom management preferences were also influenced by patients’ desire to maintain a sense of control at the end of life. Family-based care is culturally accepted, and indeed expected, to achieve a peaceful death. Healthcare professionals experienced challenges in providing sensitive symptom management given their unfamiliarity with the religious needs of Muslims.

**Conclusions:**

Co-design research methods are essential to better understand care priorities within diverse Muslim communities. Meaningful collaboration among patients, families and healthcare professionals is necessary to identify mutually acceptable and beneficial approaches to promote culturally and religiously sensitive end-of-life symptom management.

**STRENGTHS AND LIMITATIONS OF THIS STUDY**

- The search strategy covered 10 databases and included hand-searching to ensure broad literature coverage.
- The review protocol was registered on International Prospective Register of Systematic Reviews (PROSPERO) to promote transparency and reproducibility.
- The appraisal of included studies using Gough’s weight of evidence by two reviewers aided the robustness of the synthesis.
- Only peer-reviewed primary empirical studies were included.
- The exclusion of grey literature limited the scope of insights, potentially omitting valuable non-peer-reviewed contributions.

## Introduction

 Death and dying is a universal experience, yet views, needs and expectations regarding end-of-life symptom management care can vary and are influenced by religion and culture.^1^,^3^ Muslims represent the world’s fastest-growing religious group, comprising over a quarter of the global population.^4^ In Europe, Muslims make up >10% of the population in at least 10 countries.^5^ In the UK, Islam is the second largest religion after Christianity, with Muslims accounting for 6% of the national population.^6^ Reflecting broader demographic trends, the proportion of Muslims in the UK aged 65 and older is projected to rise from 4% in 2011 to 10% by 2036, reaching approximately half a million individuals.^6^ As the Muslim population ages, chronic disease morbidity and mortality underscore their increasing need for end-of-life care.^4^

End-of-life care aims to provide people typically in their last year of life with physical, psychosocial and spiritual support to help them live as comfortably as possible.^7^ Poorly managed symptoms at the end of life cause significant distress for all involved.^7^,^9^ Timely and effective symptom management is an essential component of end-of-life care and a priority for patients, family carers and care providers.^10^,^12^ For many Muslims, religious identity becomes especially salient at the end of life, shaping their care needs and decisions.^13 14^ For instance, some Muslim patients may decline medications that can cause sedation to preserve the ability to perform daily prayers or recite the *Shahada* (declaration of faith) at the time of death.^15^ Variable views of pain as a means of spiritual purification may influence attitudes toward using pain medications, with some individuals choosing to endure pain as a form of atonement of sins while others may have no concerns with using analgesics.^15 16^ Such diverse perspectives highlight the importance of culturally and religiously sensitive approaches to symptom management that respect patient and family beliefs and support informed, shared decision-making.

End-of-life care services in non-Muslim majority countries are often inadequately equipped to meet the needs of Muslim communities. For instance, Muslims in the USA report concerns about maintaining dignity at the end of life, navigating language barriers, and receiving religiously and culturally sensitive care from professionals who are unfamiliar with their faith-based traditions.^17^ In the UK, evidence suggests unmet end-of-life symptom management needs among Muslims.^3 18^ Studying the views and preferences of Muslims regarding end-of-life symptom management offers a valuable lens into how religion and culture shape experiences of death and dying and how services can meet diverse needs.

### Aim

This systematic review synthesises published evidence on Muslims’ perspectives and preferences regarding end-of-life symptom management to inform practice and research priorities.

### Review questions

This review attempts to address the following questions regarding end-of-life symptom management for Muslims living in the British Isles:

1. What are the perspectives and preferences of patients?
2. What are the perspectives and preferences of family carers?
3. What are the perspectives of healthcare professionals providing end-of-life care?
4. What is the role of faith and culture in shaping perspectives and preferences?

## Methods

A systematic review and narrative synthesis were conducted.^19 20^ A search strategy was developed in collaboration with a specialist information technologist (I.K.) and applied across MEDLINE, EMBASE, CINAHL, PsycINFO, Web of Science, ASSIA, The Cochrane Library and Global Health to capture relevant papers published between 1 January 1994 and 10 July 2024 (online supplemental document 1). The search strategy in MEDLINE is detailed in box 1. Manual searches were also conducted in the *Journal of Palliative Medicine* and the *British Medical Journal of Supportive & Palliative Care*. Reference searches of all included papers were undertaken.

Box 1 **Medline Search Strategy**

Ovid MEDLINE[R] and Epub Ahead of Print, In-Process, In-Data-Review & Other Non-Indexed Citations, Daily and Versions <1946 to January 24, 2024>

1. exp Terminal Care/ or exp Palliative Care/ or exp "Hospice and Palliative Care Nursing"/ or exp death/ or exp Palliative Medicine/ or exp Terminally Ill/ or [[end adj2 life] or [[final* or last*] adj1 [hour* or day* or minute* or week* or month* or year* or moment*]] or palliat* or terminal* or [end adj stage] or dying or [body adj2 [shutdown or shut* down or deteriorat*]] or deathbed or "pain management" or "hospice care" or "palliative medicine" or "symptom management" or "comfort care" or "incurable disease?" or "incurable condition?" or "noncurable disease?" or "noncurable condition?"].ti,ab,kw,kf. 1041610
2. exp islam/ or "religion and medicine"/ or "Transients and Migrants"/ or [muslim* or islam* or Sunni or Shia or Whabbi or Salafi or Berelvi or Sufi or Deobandi or mohammedanism or faith* or ethnic* or religio* or spirit* or migrant* or immigrant* or "expatriat*" or "refugee*"].ti,ab,kw,kf. 361196
3. exp Health Personnel/ or ["health* professional*" or nurs* or doctor* or physician* or clinician*].ti,ab,kw,kf. 1794529
4. exp United Kingdom/ or [gb or "g.b." or britain* or [british* not "british columbia"] or uk or "u.k." or united kingdom* or [england* not "new england"] or northern ireland* or northern irish* or scotland* or scottish* or [[wales or "south wales"] not "new south wales"] or welsh*].ti,ab,kw,kf,in. 2231756
5. [europ* or france* or french or spain* or spanish or german* or italy* or italian* or denmark* or danish or norway* or norwegian or sweden* or swedish or austria* or russia* or poland or polish or ukrain* or romania* or netherland* or dutch* or belgium* or belgic or belgian or czech* or greece* or greek or portugal* or portuguese or hungary* or hungarian* or belarus* or austria* or switzerland* or swiss or serbia* or bulgaria* or slovakia* or finland* or finnish or croatia* or moldova* or albania* or lithuania* or slovenia* or latvia* or estonia* or Luxembourg* or montenegr* or malta* or maltese or iceland* or icelandic or andorra* or Liechtenstein or monaco* or monegasques or "republic of ireland*" or eire or irish or herzegovina or Bosnia* or macedonia* or kosov* or "san marino*" or sammarinese or "holy see" or cypr* or Vatican city or sicily* or sicilian or gibralta* or scandinav* or Balkan* or Georgia* or Turkey or turkish or Kazakhstan or kazakh* or Azerbaijan*].ti,ab,kw,kf,in,jw. 8368666
6. europe/ or european alpine region/ or andorra/ or austria/ or balkan peninsula/ or belgium/ or europe, eastern/ or albania/ or baltic states/ or estonia/ or latvia/ or lithuania/ or "bosnia and herzegovina"/ or bulgaria/ or croatia/ or czech republic/ or hungary/ or kosovo/ or moldova/ or montenegro/ or poland/ or "republic of belarus"/ or "republic of north macedonia"/ or romania/ or russia/ or serbia/ or slovakia/ or slovenia/ or ukraine/ or france/ or germany/ or gibraltar/ or united kingdom/ or greece/ or ireland/ or italy/ or liechtenstein/ or luxembourg/ or mediterranean region/ or mediterranean islands/ or cyprus/ or malta/ or sicily/ or monaco/ or netherlands/ or portugal/ or san marino/ or "scandinavian and nordic countries"/ or denmark/ or finland/ or iceland/ or norway/ or sweden/ or spain/ or switzerland/ or vatican city/ or exp "Georgia [republic]"/ or exp turkey/ or exp Kazakhstan/ or exp Azerbaijan/ 1396135
7. 4 or 5 or 6 10355178
8. 1 and 2 and 3 and 7 1618

All identified papers were independently screened by two researchers (J.M. and K.S.) using a systematic review management platform (Rayyan) against the inclusion and exclusion criteria detailed in table 1. Initially, this systematic review was designed to focus on Muslims living in the UK and Europe, non-Muslim majority areas that host sizeable Muslim communities. However, due to the unequal spread of papers retrieved across regions, while focusing only on English-language publications, providing a comprehensive image of Muslim population experiences across the UK and Europe was not feasible. Consequently, the target population was redefined to Muslims living in the British Isles: Great Britain and the Republic of Ireland.

**Table 1:** Paper inclusion and exclusion criteria

Inclusion criteria	Exclusion criteria
The paper is about Muslim communities’ views, experiences and/or preferences regarding EoL symptom management.	The paper is about non-Muslim communities and/or euthanasia or assisted suicide. (Relevant papers with findings about Muslim and non-Muslim communities were included, but only findings pertinent to Muslim communities were extracted).
The paper is about Muslim communities living in the British Isles.	The paper is about Muslim communities living in the USA, Asia, Africa, Australia or Europe.
The paper is a peer-reviewed, primary empirical research or a conference abstract.	The paper is considered grey literature (ie, expert opinion, policy, unpublished audit, service evaluation or a review, editorial or a book that does not present new empirical research).
The full text of the paper is in English.	The full text of the paper is not in English.

A review-specific form was designed and used to extract data from all included papers, capturing publication details, study aims, participants, methods and results relevant to each of the four review questions (online supplemental document 2). All data extraction was completed by JM and double-checked for accuracy and comprehensiveness independently by KS.

An inductive narrative synthesis consisting of three steps was used to analyse tabulated findings: (1) conducting a preliminary synthesis of findings, (2) exploring relationships between studies and (3) assessing the robustness of the synthesis. A preliminary thematic analysis was conducted by JM, an early-career clinical academic nurse and Muslim, who combined results from across studies.^21^ NVivo software (version 14) was used to facilitate line-by-line descriptive coding of all review-specific findings, which resulted in 81 codes. Similar codes were combined to generate descriptive themes, and analytical themes were then identified in relation to each review question. Subsequently, a cross-study synthesis was conducted to explore the sources of homogenous and heterogenous findings across the included papers, with consideration to study sampling techniques, participant characteristics and methodological approaches. To aid the interpretative synthesis, findings were discussed and revised through reflexive discussions with KS and BB, with the positionality of a Christian international medical student and a clinical academic community nurse and Christian, respectively. The final synthesis was checked against the primary studies to ensure it was robust and grounded in the data.

This systematic review protocol was registered with PROSPERO (CRD42024497084). The Preferred Reporting Items for Systematic Reviews and Meta-Analyses guidelines were followed.^22^

Gough’s Weight of Evidence (WoE) framework was used to enhance the robustness of the synthesis by appraising each included paper in terms of its internal validity, appropriateness of methods and contribution to the research aim. Table 2 describes the dimensions of Gough’s WoE with respect to each component, its definition and the scoring approach. Each included paper was scored independently by two researchers (JM and KS) to enable a consistent appraisal approach and facilitate consensus on its contribution to answer the review questions. Discrepancies in screening and scoring decisions were discussed, and consensus was achieved.

**Table 2:** Gough’s weight of evidence framework

Component	Definition	Scoring approach(3=High, 2=Medium, 1=Low)
Weight of evidence A	Non-review-specific component to judge the coherence of the evidence.	Papers were assessed for the clarity of their aims, consistency of their methods, strengths and limitations of their methods and the relevance and proportionality of their findings to their methods. Assessments were informed by the JBI checklist across qualitative papers and the BMJ Critical Appraisal Checklist for a Questionnaire Study across surveys.
Weight of evidence B	Review-specific component to judge the appropriateness of the form of evidence for answering the review questions.	Papers that used interviews, observations and/or focus groups to capture preferences and perspectives of patients/families/HCPs or the impact of faith and culture on them were scored 3. Papers that used surveys to capture preferences and perspectives of patients/families/HCPs or the impact of faith and culture on them were scored 2. Papers that used secondary perspectives or descriptive audits to capture preferences of Muslim patients and families or the impact of faith and culture on them were scored 1.
Weight of evidence C	Review-specific component to judge the relevance of the evidence to the review questions	Papers were scored 3 if they were considered highly relevant to the review question/s by both researchers, 2 if moderately relevant and 1 if slightly relevant
Weight of evidence D	Overall assessment of the extent that a paper contributes evidence to answering a review question	Sum of WoE A, B and C.

HCP, healthcare professional; JBI, Joana Briggs Institute; WoE, weight of evidence.

## Results

**Figure 1:**
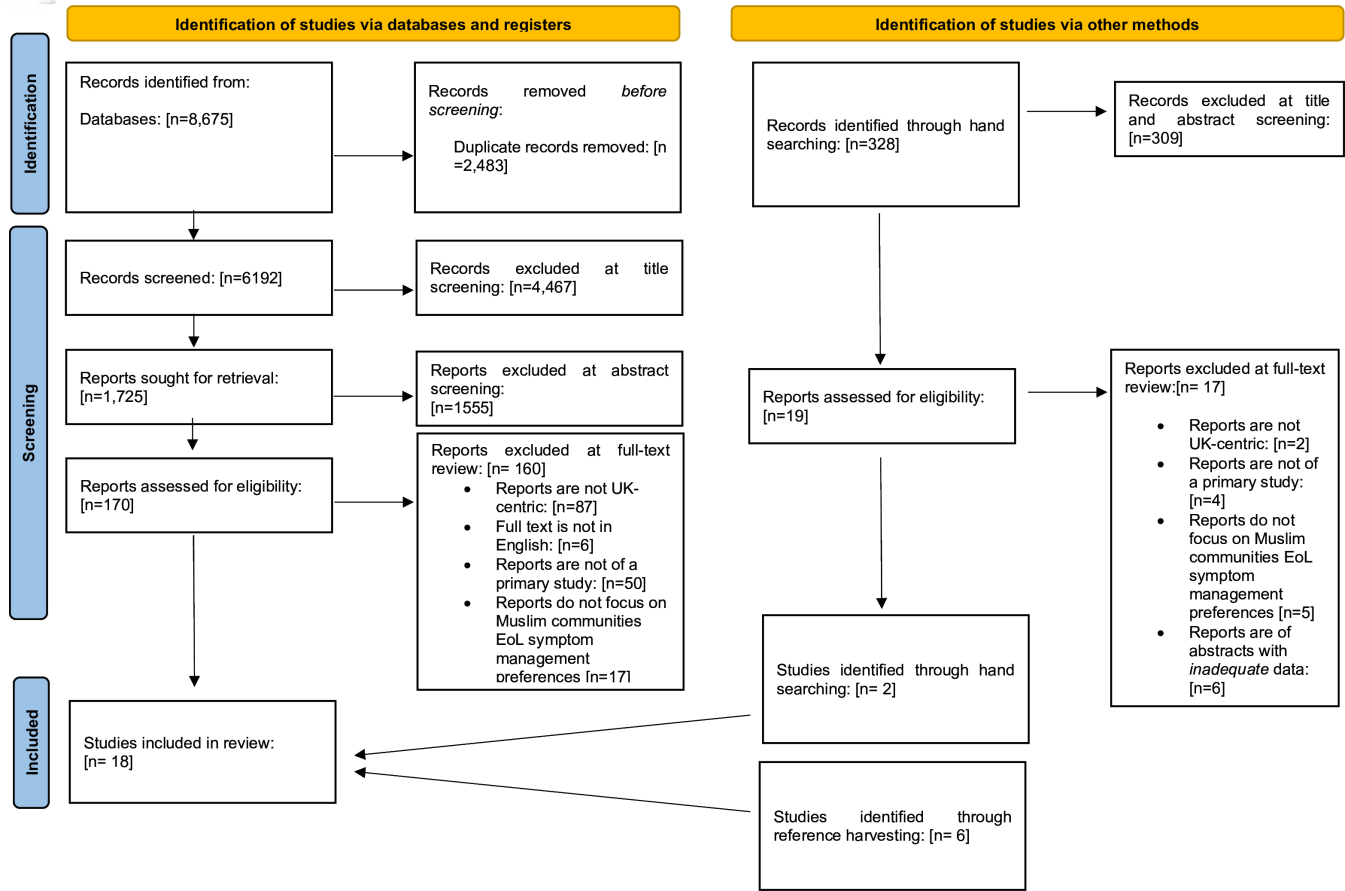
Preferred Reporting Items for Systematic Reviews and Meta-Analyses chart. EoL: end of life

The paper identification and screening process is summarised in figure 1. Database searches identified 8675 records with an additional 328 retrieved from manual journal searches. A total of 18 eligible papers, reporting on 17 studies, were included in the synthesis: 16 qualitative studies, 1 quantitative and 1 mixed-methods study. One study was reported in two papers.^23 24^ As each presented different findings, they were treated as two individual units in the synthesis. 17 papers reported on study populations in the UK and 1 in the Republic of Ireland. A summary of the included papers and their WoE scores is provided in online supplemental material 3. 11 papers were rated as WoE medium and 7 as WoE high. Published paper methods included questionnaires (n=3) and observations of healthcare professionals (n=1), qualitative interviews with patients and/or family carers (n=7), healthcare professionals (n=2) or a combination of stakeholders (n=5). Patient and family carer participants were identified as being predominantly from South Asian heritage, whereas healthcare professional participants included members of both the South Asian and White British communities.

### What are the perspectives and preferences of patients?

Six papers reported on patient perspectives, two rated as WoE medium and four high.^12 23 25^,^27^ Only one paper^25^ focused on patients’ end-of-life symptom control management views and preferences in detail; the other papers provided brief references contextualised within wider palliative care experiences. Two overarching themes were identified.

#### Being in control while receiving symptom management support

Muslim patients’ perspectives on end-of-life symptom management often centred on the desire to maintain control in ways that aligned with their religious and cultural values while achieving physical relief of symptoms.^1 25^ This included managing symptoms in a manner that allowed them to remain mentally alert and physically capable of performing essential religious duties, such as daily prayers and the recitation of the *Shahada* at the time of death.^1 23 25^ In a study by Samanta *et al*, one patient expressed, “We, as Muslims, want to be alert for as long as possible in order to read our Shahada”.^1^

#### Receiving end-of-life care via a Muslim-centred lens

Patients felt reassured when they spoke with healthcare professionals who understood their culture and religious norms, as they felt supported in a way that met their symptom management preferences and needs.^26^ However, they often felt that their end-of-life symptom management and other care preferences were inadequately met by the current healthcare system because of limited resources and inflexible institutional-based care.^27^ By observing how healthcare professionals address patients’ religious preferences, Pentaris found that the current British healthcare system responds to religious needs via a Christian-centred lens, which threatens to invalidate non-Christian religious identities.^2^ Findings collectively highlighted a dissonance between the needs of Muslim patients and available end-of-life care services, rooted in a broader structural issue, namely, the British healthcare system’s tendency to operate within a Christian-centric framework, which contributed to marginalising non-Christian needs.

### What are the perspectives and preferences of family carers?

Eight papers reported on family carer perspectives and preferences regarding symptom management, six rated as WoE medium and two high.^2426^,^32^ One theme was identified.

#### Family duty to advocate for sensitive care

Family carers expressed a deep concern that sub-optimal end-of-life care and symptom management would be provided to their loved ones if they were not present at the bedside.^24^ They voiced apprehension about leaving their loved ones in the ‘(un)care of others’, pointing to a significant lack of trust in the healthcare system.^27^ This mistrust often stemmed from perceived inadequacies in the provision of religiously and culturally sensitive care.^28 29^ Thus, they perceived their involvement as a full-time crucial duty to ensure that their loved ones’ care and symptom management remained aligned with religious and cultural expectations and often played a key role in communicating with healthcare professionals.^26 30 31^

Differences in family carers’ experiences among the included studies highlight how their involvement in symptom management care varied between community and institution-based (ie, hospice or hospital) care settings. In the community, family carers often felt a strong sense of empowerment and responsibility, as this setting allowed for a more personalised environment where they could directly influence the end-of-life care process to ensure religious and cultural needs were met. In contrast, hospital and hospice settings presented more pronounced systemic limitations, such as a lack of halal food, interpreters or culturally aware staff. In these settings, carers frequently assumed the role of primary advocates to ensure the delivery of sensitive care. Family carers often saw it as their role and duty to fill critical gaps in sensitive care.^24 26 27 30 31^ During the COVID-19 pandemic, this responsibility was often devolved to institution-based faith leaders (ie, chaplains) to provide such support because of visitation restrictions.^32^

### What are the perspectives of healthcare professionals providing end-of-life care?

Six papers reported on the perspectives of healthcare professionals providing care to Muslim communities, four rated as WoE medium and two high.^2933^,^37^ Two themes were identified.

#### Uncertainty in the presence of unfamiliarity

Overall, many healthcare professionals reported being uncertain of how to best provide religiously and culturally sensitive end-of-life symptom management for members of Muslim communities.^2933^,^35^ Hospice nurses expressed their unfamiliarity with the specific end-of-life symptom management and spiritual care needs of Muslims.^33^ They voiced concerns about how to adapt their usual care and shared how they are constantly trying to avoid causing offence.^33^ In addition to language barriers, a ‘standing back’ approach among healthcare professionals, characterised by expecting patients and families to take initiative and express their care perspectives and preferences, was found to exacerbate existing uncertainties.^35 36^ Healthcare professionals acknowledged that they tended to focus on practical end-of-life care arrangements (eg, where people want to die) or clinical aspects (eg, do not resuscitate decisions) rather than taking a holistic approach, despite local and national initiatives and training to support the integration of emotional, spiritual, cultural and social dimensions of end-of-life care.^37^,^39^

#### Collaborating to provide sensitive care

Among the included studies, healthcare professionals’ perceptions of the centrality and primacy of religion and culture among Muslim communities varied. Some hospice nurses expressed their concerns that patients could potentially suffer because of their religious beliefs, particularly due to a perceived reluctance to accept pain medications.^33^ Other healthcare professionals such as community nurses reported admiring family support among Muslims, yet often felt pressured to comply with families’ wishes rather than what the nurse identified as care priorities.^35 36^ Collaboration between the patient’s family and healthcare professionals was therefore identified essential to the effective provision of culturally sensitive end-of-life care.^35^

### What is the role of faith and culture in shaping perspectives and preferences?

Nine papers reported on the role of faith and culture in shaping perspectives and preferences, seven rated as WoE medium and two high.^2 25 27 30 33 34 36 37 40^ One theme was identified.

#### Redefining the concept of ‘good death’

Pentaris et al. and Moss et al. concluded that the concept of a ‘good death’ in the UK is often framed through a White British, and predominantly Christian, lens.^2 37^ This framing tends to overlook the cultural and religious nuances that shape end-of-life experiences for ethnic minority communities, including Muslims.^2 37^ Perceptions of what constitutes a ‘good death,’ along with preferences for symptom management, were found to be significantly influenced by faith and culture.^25 27 30 33 36 40^ For many Muslim patients and families, the primary concern is to ensure that the dying process and the use of symptom management medications align with, and do not impede, their religious and spiritual commitments. Suleman reported that for most Muslim individuals, a ‘good death’ is one that reflects a strong enduring belief in God and an attitude of patience and gratitude; as one patient shared, ‘To die well is to leave this world with steadfastness in faith and belief in God, underlined by patience and gratefulness’.^34^

## Discussion

### Summary of findings

This systematic review identified the following main findings:

1. For many Muslims, the primary concern is to ensure that the dying process and the use of symptom management medications align with their religious commitments.
2. Family carers perceived their involvement as a full-time duty to ensure that end-of-life symptom management remains aligned with religious and cultural expectations.
3. Healthcare professionals are uncertain about how to provide religiously and culturally sensitive care to Muslim patients.
4. What constitutes a good death and appropriate symptom management is shaped by faith and culture.

This review identified common facets that mirrored international studies on Muslim communities’ end-of-life care experiences in non-Muslim majority countries, including the importance of aligning symptom management with faith and culture and recurrent gaps in the delivery of sensitive end-of-life care. For instance, in the USA, healthcare professionals reported a lack of awareness regarding Muslim teachings and traditions related to end-of-life care.^17^ In the Netherlands, family involvement at the bedside was identified as both a duty and an essential component in ensuring the provision of culturally sensitive end-of-life care.^4^

Our findings also concur with end-of-life care views and experiences of other ethnic minority groups. Limited confidence and trust in healthcare services and professionals to provide culturally and religiously appropriate care are common issues identified.^41^ In a report on end-of-life care for black, Asian and minority ethnic groups in the UK, Marie Curie also highlighted the lack of sensitivity to patients’ cultural and religious differences, inadequate interpretation services and the influence of Western values on current end-of-life care models.^42^ Additionally, in a systematic review exploring end-of-life care views and needs of Gypsy, Traveller and Roma communities in the UK, Dixon et al. identified that extended families felt it was their duty to be present at the bedside and be involved in medical decision-making, and healthcare professionals voiced unfamiliarity with how to cope with these cultural needs and provide sensitive end-of-life care.^43^ Facilitating shared decision making by involving family members and other relevant stakeholders has been shown to increase prognostic awareness among patients by encouraging discussions with patients about their values and the framing of their conditions.^44^

These similarities across findings underscore the critical need for healthcare systems to recognise and address the cultural and religious diversity within the populations they serve, particularly at the end of life. Our findings and the wider emerging research highlight the shared challenges faced by Muslim communities in non-Muslim majority countries and other minority ethnic groups, emphasising the importance of culturally and religiously sensitive approaches to improve end-of-life care for ethnically diverse populations.

Our comprehensive search strategy used 10 databases, ensuring a broad range of relevant studies across healthcare settings. This review highlights the significant scarcity of research on Muslims’ end-of-life care experiences, particularly the intersection of cultural, religious and medical perspectives that are crucial to understanding their symptom management preferences. It has revealed critical gaps in addressing the unique challenges faced by Muslim communities at the end of life, including the influence of faith and religious traditions on care decisions. Only two papers^25 30^ in this review focused on patients’ end-of-life symptom control management views and preferences in detail, with the other papers providing brief references contextualised within wider end-of-life care experiences, evidencing that symptom management preferences cannot be separated from other end-of-life care needs. The findings underscore the urgent need for high-quality research to ensure that the specific needs of Muslim patients and their families are understood and effectively integrated into healthcare practices and national policies, particularly in non-Muslim majority countries.

A potential limitation of our thematic analysis was that it was conducted primarily by one member of the team. However, to aid reflexivity and the interpretative synthesis, initial insights, codes and themes were discussed and revised through discussions with two other members of the team with different positionalities and cultural backgrounds; we all read the primary studies and were familiar with the methods and findings. The included studies were re-reviewed to check the robustness of the final synthesis and ensure analytical themes were grounded in the primary data.

Corresponding to the limitations reported in the included papers, our review findings are not representative of the full diversity of Muslim communities in the British Isles, as studies primarily focused on South Asian Muslims. Although South Asian Muslims constitute more than two-thirds of Muslim populations in the UK and represent the largest ethnic group in the British Isles, they are only one of many Muslim ethnicities and do not serve as an accurate proxy for the diverse religious and cultural backgrounds within the Muslim faith.^45^ Our findings do not necessarily reflect nuances of end-of-life perspectives and preferences within and across Muslim communities in the UK, as differences in religious practices between generations and Muslim sects may also influence perspectives and preferences.^34^ Additionally, the included studies primarily focused on older adults and terminally ill patients who were approaching the end of life but were not receiving care during the final weeks or days of life. As perspectives on pain and symptom management may evolve closer to death, our findings may not fully reflect the experiences of patients in the final stages of life.

Meaningful collaboration between patients, families, faith leaders and healthcare professionals is necessary to identify mutually acceptable and effective ways to provide sensitive end-of-life care and symptom control input for Muslims. A successful example of such collaboration can be seen through the recent Muslim Council of Britain’s facilitated partnerships between hospices and local faith-based organisations to disseminate education programmes about end-of-life services to Muslim communities. This approach enables healthcare professionals to gain familiarity with religious and cultural commitments and needs of Muslims and foster trust between healthcare services, faith leaders and Muslim communities.^18^ Such collaborative approaches could help reduce the incidence of unmanaged pain, alongside reducing the uncertainty among healthcare professionals when providing religiously and culturally sensitive care.

Equally important is the integration of faith-welcoming and culture-welcoming practices, such as employing a diverse workforce, providing interpreter services, creating prayer spaces in institutional end-of-life care settings and having access to Muslim chaplains.^17^ These measures can facilitate engagement of Muslims with end-of-life care services, enhance communication of their symptom control needs and preferences and create a more inclusive healthcare environment. Muslim chaplains can play a vital role by building trust in institutional care settings and providing an in-depth understanding of Islam to healthcare professional colleagues.^17^

Efforts are also needed to improve the representativeness of Muslim communities in end-of-life care research. Considering the heterogeneity among Muslims and the consequent effect on their perspectives and preferences, it remains imperative that these cultural and religious diversities are reflected and accounted for within end-of-life care research. Additionally, as perspectives and preferences can change as dying unfolds, further qualitative research is necessary to understand and convey patients’ potentially shifting views and needs. Co-design research methods have great potential for meaningfully engaging with patients, carers, community members, faith leaders and healthcare professionals to identify issues and possible solutions to improve the delivery of sensitive end-of-life care.^46 47^

## Conclusion

This systematic review identified that aligning religion, culture and end-of-life symptom management is a priority for members of Muslim communities. However, patients and families are concerned about the abilities of current end-of-life care services in meeting their needs. The perspectives and priorities within the diverse range of Muslim communities within the UK remain underexplored. Healthcare professionals face challenges in providing sensitive symptom management owing to their unfamiliarity with religiously informed care preferences. Recommendations for research and practice include employing co-design methods to better understand care priorities within diverse Muslim communities and fostering meaningful collaboration between patients, families, faith leaders and healthcare professionals to identify mutually acceptable and beneficial approaches for delivering culturally and religiously sensitive end-of-life symptom management.

## Supplementary material

10.1136/bmjopen-2025-108877 online supplemental file 1

10.1136/bmjopen-2025-108877 online supplemental file 2

10.1136/bmjopen-2025-108877 online supplemental file 3

## Data Availability

All data relevant to the study are included in the article or uploaded as supplementary information.
