# Supplementary material for: Muslim communities’ perspectives and preferences regarding end-of-life symptom management: a systematic review and narrative synthesis
Source: BMJ Open. 2026 Jan 14;16(1):e108877. doi: 10.1136/bmjopen-2025-108877 (PMC12815058; doi:10.1136/bmjopen-2025-108877)
Supplement: online supplemental file 1 [file bmjopen-16-1-s001.docx]

**Muslim Communities Perspectives and Preference Regarding End-of-life Symptom Management**

|  | 25 January 2024 | New articles at 7 October 2024 |
| --- | --- | --- |
| Medline via Ovid | 1618 | 131 |
| Embase via Ovid | 2111 | 127 |
| CINAHL via Ebsco | 662 | 28 |
| Psycinfo via Ebsco | 2066 | 41 |
| Web of science core collection | 911 | 55 |
| ASSIA via Proquest | 916 | 159 |
| Cochrane Library | 88 | 3 |
| Global Health via Ebsco | 75 | 1 |
| Total | 8447 | 545 |
| Total deduplicated | 6164 | 413 |

Searches run 25 January 2024, updated 7 October 2024

No language limit, no publication date limit

# Medline

# Ovid MEDLINE(R) and Epub Ahead of Print, In-Process, In-Data-Review & Other Non-Indexed Citations, Daily and Versions <1946 to January 24, 2024>

# 1 exp Terminal Care/ or exp Palliative Care/ or exp "Hospice and Palliative Care Nursing"/ or exp death/ or exp Palliative Medicine/ or exp Terminally Ill/ or ((end adj2 life) or ((final* or last*) adj1 (hour* or day* or minute* or week* or month* or year* or moment*)) or palliat* or terminal* or (end adj stage) or dying or (body adj2 (shutdown or shut* down or deteriorat*)) or deathbed or "pain management" or "hospice care" or "palliative medicine" or "symptom management" or "comfort care" or "incurable disease?" or "incurable condition?" or "noncurable disease?" or "noncurable condition?").ti,ab,kw,kf. 1041610

# 2 exp islam/ or "religion and medicine"/ or "Transients and Migrants"/ or (muslim* or islam* or Sunni or Shia or Whabbi or Salafi or Berelvi or Sufi or Deobandi or mohammedanism or faith* or ethnic* or religio* or spirit* or migrant* or immigrant* or "expatriat*" or "refugee*").ti,ab,kw,kf. 361196

# 3 exp Health Personnel/ or ("health* professional*" or nurs* or doctor* or physician* or clinician*).ti,ab,kw,kf. 1794529

# 4 exp United Kingdom/ or (gb or "g.b." or britain* or (british* not "british columbia") or uk or "u.k." or united kingdom* or (england* not "new england") or northern ireland* or northern irish* or scotland* or scottish* or ((wales or "south wales") not "new south wales") or welsh*).ti,ab,kw,kf,in. 2231756

# 5 (europ* or france* or french or spain* or spanish or german* or italy* or italian* or denmark* or danish or norway* or norwegian or sweden* or swedish or austria* or russia* or poland or polish or ukrain* or romania* or netherland* or dutch* or belgium* or belgic or belgian or czech* or greece* or greek or portugal* or portuguese or hungary* or hungarian* or belarus* or austria* or switzerland* or swiss or serbia* or bulgaria* or slovakia* or finland* or finnish or croatia* or moldova* or albania* or lithuania* or slovenia* or latvia* or estonia* or Luxembourg* or montenegr* or malta* or maltese or iceland* or icelandic or andorra* or Liechtenstein or monaco* or monegasques or "republic of ireland*" or eire or irish or herzegovina or Bosnia* or macedonia* or kosov* or "san marino*" or sammarinese or "holy see" or cypr* or Vatican city or sicily* or sicilian or gibralta* or scandinav* or Balkan* or Georgia* or Turkey or turkish or Kazakhstan or kazakh* or Azerbaijan*).ti,ab,kw,kf,in,jw. 8368666

# 6 europe/ or european alpine region/ or andorra/ or austria/ or balkan peninsula/ or belgium/ or europe, eastern/ or albania/ or baltic states/ or estonia/ or latvia/ or lithuania/ or "bosnia and herzegovina"/ or bulgaria/ or croatia/ or czech republic/ or hungary/ or kosovo/ or moldova/ or montenegro/ or poland/ or "republic of belarus"/ or "republic of north macedonia"/ or romania/ or russia/ or serbia/ or slovakia/ or slovenia/ or ukraine/ or france/ or germany/ or gibraltar/ or united kingdom/ or greece/ or ireland/ or italy/ or liechtenstein/ or luxembourg/ or mediterranean region/ or mediterranean islands/ or cyprus/ or malta/ or sicily/ or monaco/ or netherlands/ or portugal/ or san marino/ or "scandinavian and nordic countries"/ or denmark/ or finland/ or iceland/ or norway/ or sweden/ or spain/ or switzerland/ or vatican city/ or exp "Georgia (republic)"/ or exp turkey/ or exp Kazakhstan/ or exp Azerbaijan/ 1396135

# 7 4 or 5 or 6 10355178

# 8 1 and 2 and 3 and 7 1618

# Embase

Embase <1974 to 2024 January 24>

1 exp *Terminal Care/ or exp *Palliative therapy/ or exp *palliative nursing/ or exp *palliative treatment/ or exp *hospice care/ or exp *hospice/ or exp *Terminally Ill Patient/ or exp *dying/ or ((end adj2 life) or ((final* or last*) adj1 (hour* or day* or minute* or week* or month* or moment*)) or palliat* or terminal* or (end adj stage) or dying or (body adj2 (shutdown or shut* down or deteriorat*)) or deathbed).ti,ab. 984284

2 exp *muslim/ or exp *islam/ or exp *migrant/ or exp refugee/ or exp *immigrant/ or (muslim* or islam* or Sunni or Shia or Whabbi or Salafi or Berelvi or Sufi or Deobandi or mohammedanism or faith* or ethnic* or religio* or spirit* or migrant* or immigrant* or "expatriat*" or "refugee*").ti,ab. 452453

3 exp *health practitioner/ or exp *medical personnel/ or exp *nurse/ or ("health* professional*" or nurs* or doctor* or physician* or clinician*).ti,ab. 2092978

4 exp United Kingdom/ or (gb or "g.b." or britain* or (british* not "british columbia") or uk or "u.k." or united kingdom* or (england* not "new england") or northern ireland* or northern irish* or scotland* or scottish* or ((wales or "south wales") not "new south wales") or welsh*).ti,ab,jx,in,ad. 3915341

5 (europ* or france* or french or spain* or spanish or german* or italy* or italian* or denmark* or danish or norway* or norwegian or sweden* or swedish or austria* or russia* or poland or polish or ukrain* or romania* or netherland* or dutch* or belgium* or belgic or belgian or czech* or greece* or greek or portugal* or portuguese or hungary* or hungarian* or belarus* or austria* or switzerland* or swiss or serbia* or bulgaria* or slovakia* or finland* or finnish or croatia* or moldova* or albania* or lithuania* or slovenia* or latvia* or estonia* or Luxembourg* or montenegr* or malta* or maltese or iceland* or icelandic or andorra* or Liechtenstein or monaco* or monegasques or "republic of ireland*" or eire or irish or herzegovina or Bosnia* or macedonia* or kosov* or "san marino*" or sammarinese or "holy see" or cypr* or Vatican city or sicily* or sicilian or gibralta* or scandinav* or Balkan* or Georgia* or Turkey or turkish or Kazakhstan or kazakh* or Azerbaijan*).ti,ab,jx,in,ad. 13256206

6 eastern europe/ or exp albania/ or exp azerbaijan/ or exp balkan peninsula/ or exp baltic states/ or exp "bosnia and herzegovina"/ or exp bulgaria/ or exp croatia/ or exp czech republic/ or exp "georgia (republic)"/ or exp hungary/ or exp kosovo/ or exp moldova/ or exp "montenegro (republic)"/ or exp poland/ or exp "republic of north macedonia"/ or exp romania/ or exp russian federation/ or exp slovakia/ or exp slovenia/ or western europe/ or austria/ or exp belgium/ or benelux/ or exp channel islands/ or exp france/ or exp germany/ or ireland/ or "isle of man"/ or liechtenstein/ or luxembourg/ or monaco/ or netherlands/ or exp scandinavia/ or switzerland/ or southern europe/ or exp andorra/ or exp gibraltar/ or exp greece/ or exp italy/ or exp malta/ or exp portugal/ or exp san marino/ or exp spain/ or exp vatican city state/ 1240322

7 4 or 5 or 6 16248745

8 1 and 2 and 3 and 7 2111

# CINAHL

| **#** | **Query** | **Limiters/Expanders** | **Last Run Via** | **Results** |
| --- | --- | --- | --- | --- |
| S8 | S1 AND S2 AND S3 AND S7 | Expanders - Apply equivalent subjects  Search modes - Boolean/Phrase | Interface - EBSCOhost Research Databases  Search Screen - Advanced Search  Database - CINAHL | 662 |
| S7 | S4 OR S5 OR S6 | Expanders - Apply equivalent subjects  Search modes - Boolean/Phrase | Interface - EBSCOhost Research Databases  Search Screen - Advanced Search  Database - CINAHL | 936,227 |
| S6 | (europ* or france* or french or spain* or spanish or german* or italy* or italian* or denmark* or danish or norway* or norwegian or sweden* or swedish or austria* or russia* or poland or polish or ukrain* or romania* or netherland* or dutch* or belgium* or belgic or belgian or czech* or greece* or greek or portugal* or portuguese or hungary* or hungarian* or belarus* or austria* or switzerland* or swiss or serbia* or bulgaria* or slovakia* or finland* or finnish or croatia* or moldova* or albania* or lithuania* or slovenia* or latvia* or estonia* or Luxembourg* or montenegr* or malta* or maltese or iceland* or icelandic or andorra* or Liechtenstein or monaco* or monegasques or "republic of ireland*" or eire or irish or herzegovina or Bosnia* or macedonia* or kosov* or "san marino*" or sammarinese or "holy see" or cypr* or Vatican city or sicily* or sicilian or gibralta* or scandinav* or Balkan* or Georgia* or Turkey or turkish or Kazakhstan or kazakh* or Azerbaijan*) | Expanders - Apply equivalent subjects  Search modes - Boolean/Phrase | Interface - EBSCOhost Research Databases  Search Screen - Advanced Search  Database - CINAHL | 603,878 |
| S5 | (MH "Europe+") OR (MH "Europe, Eastern+") OR (MH "Germany+") OR (MH "Mediterranean Region+") OR (MH "Scandinavia+") OR (MH "Andorra") OR (MH "Armenia") OR (MH "Austria") OR (MH "Azerbaijan") OR (MH "Belgium") OR (MH "European Union") OR (MH "France") OR (MH "Georgia (Republic)") OR (MH "Gibraltar") OR (MH "Greece") OR (MH "Iceland") OR (MH "Ireland") OR (MH "Italy") OR (MH "Liechtenstein") OR (MH "Luxembourg") OR (MH "Monaco") OR (MH "Netherlands") OR (MH "Portugal") OR (MH "San Marino") OR (MH "Spain") OR (MH "Switzerland") OR (MH "Albania") OR (MH "Baltic States") OR (MH "Bosnia-Herzegovina") OR (MH "Bulgaria") OR (MH "Byelarus") OR (MH "Croatia") OR (MH "Czech Republic") OR (MH "Hungary") OR (MH "Macedonia (Republic)") OR (MH "Moldova") OR (MH "Poland") OR (MH "Romania") OR (MH "Russia") OR (MH "Serbia") OR (MH "Slovakia") OR (MH "Slovenia") OR (MH "Ukraine") OR (MH "Yugoslavia") | Expanders - Apply equivalent subjects  Search modes - Boolean/Phrase | Interface - EBSCOhost Research Databases  Search Screen - Advanced Search  Database - CINAHL | 658,748 |
| S4 | (MH "United Kingdom+") OR (MH "Great Britain+") or TI ( (english not ((published or publication* or translat* or written or language* or speak* or literature or citation*) N5 english) ) OR AB ( (english not ((published or publication* or translat* or written or language* or speak* or literature or citation*) N5 english) ) | Expanders - Apply equivalent subjects  Search modes - Boolean/Phrase | Interface - EBSCOhost Research Databases  Search Screen - Advanced Search  Database - CINAHL | 340,646 |
| S3 | TI("health* professional*" or nurs* or doctor* or physician* or clinician*) or AB("health* professional*" or nurs* or doctor* or physician* or clinician*) or (MH "Health Personnel+") OR (MH "Nurses+") OR (MH "Medical Staff+") OR (MH "Physicians+") OR (MH "Allied Health Personnel+") OR (MH "Expert Clinicians+") OR (MH "Mental Health Personnel+") | Expanders - Apply equivalent subjects  Search modes - Boolean/Phrase | Interface - EBSCOhost Research Databases  Search Screen - Advanced Search  Database - CINAHL | 1,333,961 |
| S2 | TI(muslim* or islam* or Sunni or Shia or Whabbi or Salafi or Berelvi or Sufi or Deobandi or mohammedanism or faith* or ethnic* or religio* or spirit* or migrant* or immigrant* or "expatriat*" or "refugee*") or AB(muslim* or islam* or Sunni or Shia or Whabbi or Salafi or Berelvi or Sufi or Deobandi or mohammedanism or faith* or ethnic* or religio* or spirit* or migrant* or immigrant* or "expatriat*" or "refugee*") or (MH "Islam") OR (MH "Immigrants+") OR (MH "Undocumented Immigrants") OR (MH "Emigration and Immigration") OR (MH "Transients and Migrants") OR (MH "Refugees+") | Expanders - Apply equivalent subjects  Search modes - Boolean/Phrase | Interface - EBSCOhost Research Databases  Search Screen - Advanced Search  Database - CINAHL | 173,724 |
| S1 | TI ((end N2 life) or ((final* or last*) N1 (hour* or day* or minute* or week* or month* or moment*)) or palliat* or terminal* or (“end stage”) or dying or (body N2 (shutdown or shut* down or deteriorat*)) or deathbed) or AB ((end N2 life) or ((final* or last*) N1 (hour* or day* or minute* or week* or month* or moment*)) or palliat* or terminal* or (“end stage”) or dying or (body N2 (shutdown or shut* down or deteriorat*)) or deathbed) or (MH "Terminal Care+") OR (MH "Palliative Care") OR (MH "Hospice and Palliative Nursing") OR (MH "Hospice Patients") OR (MH "Hospices") OR (MH "Hospice Care") OR (MH "Terminally Ill Patients") OR (MH "Death+") | Expanders - Apply equivalent subjects  Search modes - Boolean/Phrase | Interface - EBSCOhost Research Databases  Search Screen - Advanced Search  Database - CINAHL | 222,041 |

Bottom of Form

# Psycinfo

| **#** | **Query** | **Limiters/Expanders** | **Last Run Via** | **Results** |
| --- | --- | --- | --- | --- |
| S8 | S1 AND S2 AND S3 AND S7 | Expanders - Apply equivalent subjects Search modes - Boolean/Phrase | Interface - EBSCOhost Research Databases Search Screen - Basic Search Database - APA PsycInfo | 2,066 |
| S7 | S4 OR S5 OR S6 | Expanders - Apply equivalent subjects Search modes - Boolean/Phrase | Interface - EBSCOhost Research Databases Search Screen - Basic Search Database - APA PsycInfo | 5,246,603 |
| S6 | (gb or "g.b." or britain* or (british* not "british columbia") or uk or "u.k." or united kingdom* or (england* not "new england") or northern ireland* or northern irish* or scotland* or scottish* or ((wales or "south wales") not "new south wales") or welsh*) | Expanders - Apply equivalent subjects Search modes - Boolean/Phrase | Interface - EBSCOhost Research Databases Search Screen - Basic Search Database - APA PsycInfo | 566,731 |
| S5 | (europ* or france* or french or spain* or spanish or german* or italy* or italian* or denmark* or danish or norway* or norwegian or sweden* or swedish or austria* or russia* or poland or polish or ukrain* or romania* or netherland* or dutch* or belgium* or belgic or belgian or czech* or greece* or greek or portugal* or portuguese or hungary* or hungarian* or belarus* or austria* or switzerland* or swiss or serbia* or bulgaria* or slovakia* or finland* or finnish or croatia* or moldova* or albania* or lithuania* or slovenia* or latvia* or estonia* or Luxembourg* or montenegr* or malta* or maltese or iceland* or icelandic or andorra* or Liechtenstein or monaco* or monegasques or "republic of ireland*" or eire or irish or herzegovina or Bosnia* or macedonia* or kosov* or "san marino*" or sammarinese or "holy see" or cypr* or Vatican city or sicily* or sicilian or gibralta* or scandinav* or Balkan* or Georgia* or Turkey or turkish or Kazakhstan or kazakh* or Azerbaijan*) | Expanders - Apply equivalent subjects Search modes - Boolean/Phrase | Interface - EBSCOhost Research Databases Search Screen - Basic Search Database - APA PsycInfo | 1,350,652 |
| S4 | TI ( (english not ((published or publication* or translat* or written or language* or speak* or literature or citation*) N5 english) ) OR AB ( (english not ((published or publication* or translat* or written or language* or speak* or literature or citation*) N5 english) ) | Expanders - Apply equivalent subjects Search modes - Boolean/Phrase | Interface - EBSCOhost Research Databases Search Screen - Basic Search Database - APA PsycInfo | 5,213,972 |
| S3 | TI("health* professional*" or nurs* or doctor* or physician* or clinician*) or AB("health* professional*" or nurs* or doctor* or physician* or clinician*) or ((DE "Physicians" OR DE "Family Physicians" OR DE "General Practitioners" OR DE "Gynecologists" OR DE "Internists" OR DE "Neurologists" OR DE "Obstetricians" OR DE "Pathologists" OR DE "Pediatricians" OR DE "Psychiatrists" OR DE "Surgeons") OR (DE "Nurses" OR DE "Psychiatric Nurses" OR DE "Public Health Service Nurses" OR DE "School Nurses")) AND (DE "Health Personnel" OR DE "Allied Health Personnel" OR DE "Caregivers" OR DE "Medical Personnel" OR DE "Mental Health Personnel" OR DE "Mental Health Personnel" OR DE "Clinical Psychologists" OR DE "Psychiatric Hospital Staff" OR DE "Psychiatric Nurses" OR DE "Psychiatric Social Workers" OR DE "Psychiatrists" OR DE "Psychotherapists" OR DE "School Psychologists") | Expanders - Apply equivalent subjects Search modes - Boolean/Phrase | Interface - EBSCOhost Research Databases Search Screen - Basic Search Database - APA PsycInfo | 357,495 |
| S2 | TI(muslim* or islam* or Sunni or Shia or Whabbi or Salafi or Berelvi or Sufi or Deobandi or mohammedanism or faith* or ethnic* or religio* or spirit* or migrant* or immigrant* or "expatriat*" or "refugee*") or AB(muslim* or islam* or Sunni or Shia or Whabbi or Salafi or Berelvi or Sufi or Deobandi or mohammedanism or faith* or ethnic* or religio* or spirit* or migrant* or immigrant* or "expatriat*" or "refugee*") or (((DE "Muslims" OR DE "Muslim Americans") OR (DE "Islam")) OR (DE "Immigration" OR DE "Undocumented Immigration")) OR (DE "Refugees") | Expanders - Apply equivalent subjects Search modes - Boolean/Phrase | Interface - EBSCOhost Research Databases Search Screen - Basic Search Database - APA PsycInfo | 279,869 |
| S1 | TI ((end N2 life) or ((final* or last*) N1 (hour* or day* or minute* or week* or month* or moment*)) or palliat* or terminal* or (“end stage”) or dying or (body N2 (shutdown or shut* down or deteriorat*)) or deathbed) or AB ((end N2 life) or ((final* or last*) N1 (hour* or day* or minute* or week* or month* or moment*)) or palliat* or terminal* or (“end stage”) or dying or (body N2 (shutdown or shut* down or deteriorat*)) or deathbed) or ((DE "Terminally Ill Patients") OR (DE "Palliative Care")) OR (DE "Death and Dying" OR DE "Euthanasia" OR DE "Parental Death") OR (DE "Hospice") | Expanders - Apply equivalent subjects Search modes - Boolean/Phrase | Interface - EBSCOhost Research Databases Search Screen - Basic Search Database - APA PsycInfo | 104,866 |

Bottom of Form

# Web of science

# # Database: Web of Science Core Collection

# - WOS.IC: 1993 to 2024

# - WOS.CCR: 1985 to 2024

# - WOS.SCI: 1900 to 2024

# - WOS.AHCI: 1975 to 2024

# - WOS.BHCI: 2008 to 2024

# - WOS.BSCI: 2008 to 2024

# - WOS.ESCI: 2019 to 2024

# - WOS.ISTP: 1990 to 2024

# - WOS.SSCI: 1956 to 2024

# - WOS.ISSHP: 1990 to 2024

# # Searches:

# 1: TS=((end near/2 life) or ((final* or last*) near/1 (hour* or day* or minute* or week* or month* or moment*)) or palliat* or terminal* or (“end stage”) or dying or deathbed or (body near/2 deteriorat*) or (body near/2 "shut* down") or (body near/2 shutdown)) Date Run: Thu Jan 25 2024 16:59:58 GMT+0000 (Greenwich Mean Time) Results: 1750452

# 2: TS=(muslim* or islam* or Sunni or Shia or Whabbi or Salafi or Berelvi or Sufi or Deobandi or mohammedanism or faith* or ethnic* or religio* or spirit* or migrant* or immigrant* or "expatriat*" or "refugee*") Date Run: Thu Jan 25 2024 17:00:08 GMT+0000 (Greenwich Mean Time) Results: 954460

# 3: TS=("health* professional*" or nurs* or doctor* or physician* or clinician*) Date Run: Thu Jan 25 2024 17:00:16 GMT+0000 (Greenwich Mean Time) Results: 1249582

# 4: TS=(english not ((published or publication* or translat* or written or language* or speak* or literature or citation*) near/5 english)) Date Run: Thu Jan 25 2024 17:00:30 GMT+0000 (Greenwich Mean Time) Results: 199173

# 5: TS=(gb or "g.b." or britain* or (british* not "british columbia") or uk or "u.k." or “united kingdom*” or (england* not "new england") or “northern ireland*” or “northern irish*” or scotland* or scottish* or ((wales or "south wales") not "new south wales") or welsh*) Date Run: Thu Jan 25 2024 17:00:39 GMT+0000 (Greenwich Mean Time) Results: 887769

# 6: TS=(europ* or france* or french or spain* or spanish or german* or italy* or italian* or denmark* or danish or norway* or norwegian or sweden* or swedish or austria* or russia* or poland or polish or ukrain* or romania* or netherland* or dutch* or belgium* or belgic or belgian or czech* or greece* or greek or portugal* or portuguese or hungary* or hungarian* or belarus* or austria* or switzerland* or swiss or serbia* or bulgaria* or slovakia* or finland* or finnish or croatia* or moldova* or albania* or lithuania* or slovenia* or latvia* or estonia* or Luxembourg* or montenegr* or malta* or maltese or iceland* or icelandic or andorra* or Liechtenstein or monaco* or monegasques or "republic of ireland*" or eire or irish or herzegovina or Bosnia* or macedonia* or kosov* or "san marino*" or sammarinese or "holy see" or cypr* or Vatican city or sicily* or sicilian or gibralta* or scandinav* or Balkan* or Georgia* or Turkey or turkish or Kazakhstan or kazakh* or Azerbaijan*) Date Run: Thu Jan 25 2024 17:00:50 GMT+0000 (Greenwich Mean Time) Results: 4932257

# 7: #5 OR #4 OR #6 Date Run: Thu Jan 25 2024 17:00:57 GMT+0000 (Greenwich Mean Time) Results: 5798144

# 8: #1 AND #2 AND #3 AND #7 Date Run: Thu Jan 25 2024 17:01:04 GMT+0000 (Greenwich Mean Time) Results: 911

# ASSIA

| Set# | Searched for | Databases | Results |
| --- | --- | --- | --- |
| S1 | TI ((end N2 life) or ((final* or last*) N1 (hour* or day* or minute* or week* or month* or moment*)) or palliat* or terminal* or (“end stage”) or dying or (body N2 (shutdown or shut* down or deteriorat*)) or deathbed) or AB ((end N2 life) or ((final* or last*) N1 (hour* or day* or minute* or week* or month* or moment*)) or palliat* or terminal* or (“end stage”) or dying or (body N2 (shutdown or shut* down or deteriorat*)) or deathbed) | Applied Social Sciences Index & Abstracts (ASSIA) | 2255 |
| S2 | TI(muslim* or islam* or Sunni or Shia or Whabbi or Salafi or Berelvi or Sufi or Deobandi or mohammedanism or faith* or ethnic* or religio* or spirit* or migrant* or immigrant* or "expatriat*" or "refugee*") or AB(muslim* or islam* or Sunni or Shia or Whabbi or Salafi or Berelvi or Sufi or Deobandi or mohammedanism or faith* or ethnic* or religio* or spirit* or migrant* or immigrant* or "expatriat*" or "refugee*") | Applied Social Sciences Index & Abstracts (ASSIA) | 87194 |
| S3 | TI("health* professional*" or nurs* or doctor* or physician* or clinician*) or AB("health* professional*" or nurs* or doctor* or physician* or clinician*) | Applied Social Sciences Index & Abstracts (ASSIA) | 182506 |
| S4 | (gb or "g.b." or britain* or (british* not "british columbia") or uk or "u.k." or ("united kingdom" OR "united kingdoma" OR "united kingdomin" OR "united kingdoms" OR "united kingdomthe") or (england* not "new england") or ("northern ireland" OR "northern irelandin" OR "northern irelands" OR "northern irelandthe") or ("northern irish" OR "northern irishman") or scotland* or scottish* or ((wales or "south wales") not "new south wales") or welsh*) | Applied Social Sciences Index & Abstracts (ASSIA) | 468713 |
| S5 | (europ* or france* or french or spain* or spanish or german* or italy* or italian* or denmark* or danish or norway* or norwegian or sweden* or swedish or austria* or russia* or poland or polish or ukrain* or romania* or netherland* or dutch* or belgium* or belgic or belgian or czech* or greece* or greek or portugal* or portuguese or hungary* or hungarian* or belarus* or austria* or switzerland* or swiss or serbia* or bulgaria* or slovakia* or finland* or finnish or croatia* or moldova* or albania* or lithuania* or slovenia* or latvia* or estonia* or Luxembourg* or montenegr* or malta* or maltese or iceland* or icelandic or andorra* or Liechtenstein or monaco* or monegasques or "republic of ireland*" or eire or irish or herzegovina or Bosnia* or macedonia* or kosov* or ("san marino") or sammarinese or "holy see" or cypr* or Vatican city or sicily* or sicilian or gibralta* or scandinav* or Balkan* or Georgia* or Turkey or turkish or Kazakhstan or kazakh* or Azerbaijan*) | Applied Social Sciences Index & Abstracts (ASSIA) | 356115 |
| S6 | s4 or s5 | Applied Social Sciences Index & Abstracts (ASSIA) | 4913 |
| S7 | s1 and s2 and s3 and s6 | Applied Social Sciences Index & Abstracts (ASSIA) | 916 |

# Cochrane Library

Search Name:

Date Run: 26/01/2024 00:52:35

Comment:

ID Search Hits

#1 (((end near/2 life) or ((final* or last*) near/1 (hour* or day* or minute* or week* or month* or moment*)) or palliat* or terminal* or (end next stage) or dying or deathbed or (body near/2 deteriorat*) or (body near/2 (shut* next down)) or (body near/2 shutdown))):ti,ab,kw (Word variations have been searched) 57184

#2 ((muslim* or islam* or Sunni or Shia or Whabbi or Salafi or Berelvi or Sufi or Deobandi or mohammedanism or faith* or ethnic* or religio* or spirit* or migrant* or immigrant* or expatriat* or "refugee*")):ti,ab,kw (Word variations have been searched) 22219

#3 (((health* next professional*) or nurs* or doctor* or physician* or clinician*)):ti,ab,kw (Word variations have been searched) 147346

#4 (europ* or france* or french or spain* or spanish or german* or italy* or italian* or denmark* or danish or norway* or norwegian or sweden* or swedish or austria* or russia* or poland or polish or ukrain* or romania* or netherland* or dutch* or belgium* or belgic or belgian or czech* or greece* or greek or portugal* or portuguese or hungary* or hungarian* or belarus* or austria* or switzerland* or swiss or serbia* or bulgaria* or slovakia* or finland* or finnish or croatia* or moldova* or albania* or lithuania* or slovenia* or latvia* or estonia* or Luxembourg* or montenegr* or malta* or maltese or iceland* or icelandic or andorra* or Liechtenstein or monaco* or monegasques or ireland* or eire or irish or herzegovina or Bosnia* or macedonia* or kosov* or (san next marino) or sammarinese or (holy next see) or cypr* or (Vatican next city) or sicily* or sicilian or gibralta* or scandinav* or Balkan* or Georgia* or Turkey or turkish or Kazakhstan or kazakh* or Azerbaijan*):ti,ab,kw (Word variations have been searched) 158743

#5 gb or "g.b." or britain* or uk or "u.k." or "united kingdom" or england or scotland or ireland or wales 154503

#6 #1 and #2 and #3 and (#4 or #5) 90

# Global health

| **#** | **Query** | **Limiters/Expanders** | **Last Run Via** | **Results** |
| --- | --- | --- | --- | --- |
| S8 | S1 AND S2 AND S3 AND S7 | Expanders - Apply equivalent subjects Search modes - Boolean/Phrase | Interface - EBSCOhost Research Databases Search Screen - Basic Search Database - Global Health | 75 |
| S7 | S4 OR S5 OR S6 | Expanders - Apply equivalent subjects Search modes - Boolean/Phrase | Interface - EBSCOhost Research Databases Search Screen - Basic Search Database - Global Health | 1,604,049 |
| S6 | ( (english not ((published or publication* or translat* or written or language* or speak* or literature or citation*) N5 english) ) | Expanders - Apply equivalent subjects Search modes - Boolean/Phrase | Interface - EBSCOhost Research Databases Search Screen - Basic Search Database - Global Health | 14,999 |
| S5 | (europ* or france* or french or spain* or spanish or german* or italy* or italian* or denmark* or danish or norway* or norwegian or sweden* or swedish or austria* or russia* or poland or polish or ukrain* or romania* or netherland* or dutch* or belgium* or belgic or belgian or czech* or greece* or greek or portugal* or portuguese or hungary* or hungarian* or belarus* or austria* or switzerland* or swiss or serbia* or bulgaria* or slovakia* or finland* or finnish or croatia* or moldova* or albania* or lithuania* or slovenia* or latvia* or estonia* or Luxembourg* or montenegr* or malta* or maltese or iceland* or icelandic or andorra* or Liechtenstein or monaco* or monegasques or "republic of ireland*" or eire or irish or herzegovina or Bosnia* or macedonia* or kosov* or "san marino*" or sammarinese or "holy see" or cypr* or Vatican city or sicily* or sicilian or gibralta* or scandinav* or Balkan* or Georgia* or Turkey or turkish or Kazakhstan or kazakh* or Azerbaijan*) | Expanders - Apply equivalent subjects Search modes - Boolean/Phrase | Interface - EBSCOhost Research Databases Search Screen - Basic Search Database - Global Health | 1,437,626 |
| S4 | (gb or "g.b." or britain* or (british* not "british columbia") or uk or "u.k." or “united kingdom*” or (england* not "new england") or “northern ireland*” or “northern irish*” or scotland* or scottish* or ((wales or "south wales") not "new south wales") or welsh*) | Expanders - Apply equivalent subjects Search modes - Boolean/Phrase | Interface - EBSCOhost Research Databases Search Screen - Basic Search Database - Global Health | 296,174 |
| S3 | TI("health* professional*" or nurs* or doctor* or physician* or clinician*) or AB("health* professional*" or nurs* or doctor* or physician* or clinician*) | Expanders - Apply equivalent subjects Search modes - Boolean/Phrase | Interface - EBSCOhost Research Databases Search Screen - Basic Search Database - Global Health | 155,977 |
| S2 | TI(muslim* or islam* or Sunni or Shia or Whabbi or Salafi or Berelvi or Sufi or Deobandi or mohammedanism or faith* or ethnic* or religio* or spirit* or migrant* or immigrant* or "expatriat*" or "refugee*") or AB(muslim* or islam* or Sunni or Shia or Whabbi or Salafi or Berelvi or Sufi or Deobandi or mohammedanism or faith* or ethnic* or religio* or spirit* or migrant* or immigrant* or "expatriat*" or "refugee*") | Expanders - Apply equivalent subjects Search modes - Boolean/Phrase | Interface - EBSCOhost Research Databases Search Screen - Basic Search Database - Global Health | 104,090 |
| S1 | TI ((end N2 life) or ((final* or last*) N1 (hour* or day* or minute* or week* or month* or moment*)) or palliat* or terminal* or (“end stage”) or dying or (body N2 (shutdown or shut* down or deteriorat*)) or deathbed) or AB ((end N2 life) or ((final* or last*) N1 (hour* or day* or minute* or week* or month* or moment*)) or palliat* or terminal* or (“end stage”) or dying or (body N2 (shutdown or shut* down or deteriorat*)) or deathbed) | Expanders - Apply equivalent subjects Search modes - Boolean/Phrase | Interface - EBSCOhost Research Databases Search Screen - Basic Search Database - Global Health | 71,367 |

Bottom of Form
