# Supplementary material for: Muslim communities’ perspectives and preferences regarding end-of-life symptom management: a systematic review and narrative synthesis
Source: BMJ Open. 2026 Jan 14;16(1):e108877. doi: 10.1136/bmjopen-2025-108877 (PMC12815058; doi:10.1136/bmjopen-2025-108877)
Supplement: online supplemental file 3 [file bmjopen-16-1-s003.pdf]

Table 3: Summary of Included Papers

Authors and Date	Care Setting	Aim[s]	Design	Sample	Methods		Key Findings	WoE Scores
					**Type[s] of Data Collected**	**Method[s] of Data Analysis**		**A+B+C=D**
**Bajwah et.al [2021]**	Hospice, hospital, and nursing homes	To map and develop insights into the response of specialist palliative care services caring for patients and families from ethnic minority groups during the first wave of the COVID-19 pandemic	Survey	HCPs and service leads	Open-ended survey questions	Thematic analysis	• Families of patients from ethnic minority groups were particularly affected by restrictions on visiting<br>• The barriers to communication for these individuals from ethnic minorities were compounded	M L M= M
**Seale et.al [2010]**	Hospital and GP practices	To compare ethnicity and religious faith in the medical and general UK populations, and report on their associations with ethically controversial decisions	Survey	Doctors	A structured questionnaire	A multivariate analysis using logistic regression	• Independently of speciality, doctors who described themselves as nonreligious were more likely than others to report having given continuous deep sedation until death, having taken decisions, they expected or partly intended to end life, and to have discussed these decisions with patients judged to have the capacity to participate in discussions	L M M=M
**Clarke et al. [2023]**	Specialist palliative and primary	To explore the experiences and attitudes of patients and family carers from	Qualitative [Descriptive	Patients and family carers	Qualitative semi-structured interviews	Reflexive thematic analysis	• Good communication with HCPs increased the participants' understanding about their pain<br>• Most of the participants expressed some concerns and fears about pain medication including	M H H=H

	care settings	South Asian communities about pain and its management at EoL	Phenomeno logy]				apprehensions about the quantities and strength of painkillers being taken	
**Henry et.al [2016]**	Specialist palliative care inpatient unit of an Irish hospice	To gain an understanding of nurses' experiences of providing care to patients from minority ethnic groups	Qualitative	Nurses	Unstructur ed interviews	Hermeneutic phenomenolo gical approach	• There was a perception amongst nurses that patients could potentially suffer because of their religious beliefs<br>• In the context of meeting patients' religious needs, a recurrent theme across participants' accounts was that they were often feeling their way, acting slowly and carefully in the presence of uncertainty or the unfamiliar	M H H= H
**Hossain et.al [2022]**	Communit y support groups and care homes	To explore the experiences of carers of people with dementia of South Asian origin, living in the UK.	Qualitative	Family carers	In-depth face-to-face semi-structured interviews	Thematic analysis	• Family carers described how they had witnessed poor delivery of care towards their relatives<br>• When asking about advanced planning, some described how they would typically avoid discussing death and/or completion of a will due to issues related to stigma	H H L= M
**Hudson et.al [2024]**	Not reported	To explore experiences of British Muslims with palliative care needs and their families during the COVID-19 pandemic	Qualitative	Patients and family carers	Telephone interviews	Thematic analysis	• Participants felt excluded by public health messaging and government policies during the pandemic and felt they implemented without consideration<br>• Participants felt reassured when they spoke with HCPS familiar with their faith and culture	H H M= H

**Islam et.al [2023]**	GP, community care settings, secondary care, and specialist palliative care settings	To study barriers to and enablers of ethnically diverse patients, family caregivers and health-care professionals engaging in EoL care planning.	Qualitative	Bereaved family carers, HCPs, educators, and academics	Case studies, interviews, and HCP responses to the findings	Thematic analysis	• Family duty and community expectations were foregrounded in some accounts and concern about being in the "[un]care" of strangers was common<br>• Many HCPs did not see end-of-life care planning discussions as falling within their remit	M M L=M
**Somerville [2001]**	St Joseph's Hospice	To explore the experiences of carers from the Bangladeshi community and develop understanding of the carers' experiences and give them meaning	Qualitative	Family carers	In-depth semi-structured interviews	Thematic analysis	• Caring was a full-time occupation for all the participants<br>• The prime rationale for caring was the bond of love, however, responsibility and duty clearly emerged as motivation	M H M= M
**Kristiansen et.al [2014]**	Not reported	To explore the role of faith and religious identities in shaping end of life experiences in South Asian Muslims and Sikhs with life-limiting illnesses.	Qualitative	Patients, family carers, and HCP.	In-depth semi-structured interviews	Interpretive Phenomenological Analysis	• Participants stressed the importance of seeking treatment for as long as possible to enable them to maintain or regain valued social roles<br>• Hope was nurtured in social relationships and faith. At times, however, the expectations on patients to remain hopeful despite their poor prognosis and immense suffering put a tremendous burden on them	H H M= H
**Manyimo et.al [2023]**	Community	To explore the experiences of community	Qualitative	Nurses	Virtual semi-	Thematic analysis	• There was a strong mistrust and suspicion of Western medicine and a belief that it speeds up death	L H M= M

		nursing staff caring for EoL patients			structured interviews			
**Moss et.al [2023]**	Not reported	To identify the barriers that influence how South Asian communities' access and use two new palliative care services	Qualitative	HCP and other palliative care professional stakeholders	A self-completed questionnaire followed by online focus groups	Descriptive statistics [readiness scores] and reflexive thematic analysis [focus groups]	• It was felt that the prevalent view of what a 'good death' may be is based on a white British perspective, and it is not known what a 'good death' means to members of South Asian communities	L H M= M
**Owens et.al [2004]**	Home and community-based palliative care settings	To investigate the challenges faced by those trying to develop culturally competent palliative care for South Asian cancer patients in Luton, UK.	Qualitative [Phenomenology]	Patients, family carers, HCP, Muslim patient and family carers, palliative care professional stakeholders	In-depth semi-structured interviews	Thematic analysis	• Cultural competence may require a more interventionist and persistent approach to the provision of services than philosophies of palliative care usually allow	M H M= M
**Pentaris [2018]**	Hospice	To explore how professionals in end-of-life care respond to service users' religious and spiritual indicators, through the lens of religious literacy.	Qualitative [Ethnography]	HCP	Observations	Not reported	• It is suggested that healthcare practice is filtered via a Christian-centred lens, which may lead to invalidating non-Christian religious identities	M H L= M

**Samanta et.al [2018]**	Not reported	To propose a bioethical framework that accommodates religious and faith-based values of migrants at end-of-life, within the normative canvas of receiving countries	Qualitative	Self-selected members of the South Asian community	Focus-group discussions digitally recorded	Thematic analysis	• Freedom to practise rituals of religious significance at end-of-life was a shared expectation of participants<br>• There was a reluctance to accept medical treatment that preserved life in an impaired state of consciousness	H H M= H
**Suleman [2023]**	Hospice	To study the views of and experiences of Muslims in the UK about death and dying to understand the impact of different cultures, languages, and the Islamic faith on palliative and end of life care.	Qualitative	Patients, family carers, HCP, Muslim patient and family carers, palliative care professional stakeholders and Islamic scholars	Semi-structured interviews	Framework analysis	• Many Muslim patients and families described how they do not seek to evade death rather want to ensure that dying and the process of death is harmonious with their faith commitments<br>• HCPs faced challenges faced in understanding the beliefs and commitments of Muslim patients and families based on the virtue of hope, and often perceived them as manifestation of denial	M H H= H
**Venkatasalu et.al [2014]**	Community.	To explore beliefs, attitudes and expectations expressed by older South Asians living in East London about dying at home	Qualitative	Older adults from the South Asian ethnicity	Focus groups and in-depth semi-structured interviews	Constructivist grounded theory approach	• Views of home as a place where religious needs at the point of death could be met contrasted sharply with most participants' perceptions of what death might be like in hospital	H H M= H

**Venkatasalu et.al [2017]**	Hospital	To investigate older British South Asian minority ethnic patients on dying in acute hospitals	Qualitative [Grounded Theory]	Older adults from the South Asian ethnicity	Interviews and focus groups	Thematic analysis	• Participants reported that sub-optimal end-of-life care in the hospital is bound to happen if the family is not there for their loved ones during their EoL care	M H M= M
**Worth et.al [2009]**	Not reported	To examine the care experiences of South Asian Sikh and Muslim patients in Scotland with life limiting illness and their families and to understand the reasons for any difficulties with access to services and how these might be overcome.	Qualitative	Patients, family carers, and HCP.	In-depth, semi-structured interviews	Thematic analysis	• Some patients perceived the hospice as “somewhere to go to die”<br>• Planning effective end of life care was difficult when there was a lack of open discussion about dying	M H M= M
