# Supplementary material for: Muslim communities’ perspectives and preferences regarding end-of-life symptom management: a systematic review and narrative synthesis
Source: BMJ Open. 2026 Jan 14;16(1):e108877. doi: 10.1136/bmjopen-2025-108877 (PMC12815058; doi:10.1136/bmjopen-2025-108877)
Supplement: Supplementary file 4 [file bmjopen-2025-108877.reviewer_comments.pdf]

PEER REVIEW HISTORY

BMJ Open publishes all reviews undertaken for accepted manuscripts. Reviewers are asked to complete a checklist review form and are provided with free text boxes to elaborate on their assessment. These free text comments are reproduced below.

ARTICLE DETAILS

Title (Provisional)

Muslim Communities' Perspectives and Preferences Regarding End-of-Life Symptom Management: A Systematic Review and Narrative Synthesis

Authors

Mourhli, Joodi; Sosnowski, Krzysztof; Kuhn, Isla; Bowers, Ben

---

VERSION 1 - REVIEW

Reviewer 1

Name Monaco, Fabrizio

Affiliation Scientific Institute San Raffaele, Cardiothoracic and Vascular Anesthesia

Date 19-Aug-2025

COI None

I thank the Editor for allowing me an opportunity to review the manuscript 'Muslim Communities' Perspectives and Preferences Regarding End-of-Life Symptom Management: A Systematic Review and Narrative Synthesis' by Mourhli et al. submitted to 'BMJ Open'.

In this Review article, the authors sought to synthesize research evidence on Muslims' perspectives and preferences regarding end-of-life symptom management. They performed a systematic search of several databases (i.e., MEDLINE, EMBASE, CINAHL, PsycINFO, Web of Science, ASSIA, The Cochrane Library, and Global Health) and then provided a narrative synthesis of their search results. They identified 18 papers which were suitable for inclusion and found that: i) conformity between religion, culture, and end-of-life symptom management was prioritized by patients; ii) symptom management preferences were also influenced by patients' desire to maintain a sense of control at the end-of-life; iii) family-based care is culturally accepted, and indeed expected, to achieve a peaceful death; iv) healthcare professionals experienced challenges to provide sensitive symptom management due to their unfamiliarity with religious needs of Muslims. The authors concluded that co-design research methods are essential to better understand care priorities within diverse Muslim communities and that meaningful collaboration is necessary to identify mutually

acceptable and beneficial approaches to promote culturally and religiously sensitive end-of-life symptom management.

In short, this is simply a fabulous investigation, which I have read with extreme interest. I am persuaded that the presented work adds to the existing body of knowledge in this field. The manuscript is genuinely wonderfully written, and no major linguistic and/or lexical inaccuracies are present. The Figure and Table add additional value to the authors' work. The methodology is strong and robust, the PRISMA checklist was followed in detail, prior registration on PROSPERO was executed, the search strategy and study selection process is described in detail, the results are presented objectively, and the discussion constitutes a well-balanced opportunity to elaborate more on the research findings.

This expert Reviewer would nevertheless like to raise the following concerns for the authors to address to further improve their investigation, merely in order to further widen some of the hints for reflection they have presented and discussed already. Please consider the points below, in no specific order of importance.

GENERAL COMMENTS

• I believe a comprehensive Figure in the form of a 'Visual/Graphical abstract' should be added to the manuscript which provides to provide a visual representation of the study background/aims, methodology, results and implication for clinical practice.

SPECIFIC COMMENTS

• Please consider widening the scope of your 'Introduction' section by implementing some of the considerations which emerged from another recent article on this topic, i.e., PMID: 34860584.

• In your 'Discussion' section, suggest also mentioning that tensions between religious and secular identities may impact spiritual care provision in the patient population of terminally ill patients, and that research focusing on the spiritual care needs of Muslims in Israel conducted by Baddarni et al. indicated that this tension may be less relevant for Israeli Muslims. Please refer to and add citation of both i) PMID: 39093919 and ii) Baddarni K. [Spiritual Care for the Muslim Patient] Spirit Care Muslim Patient Artic Hebr 2013; 26–36.

• In your 'Discussion' section, you state that: 'Dixon et al., (2021) identified extended families felt it was their duty to be present at the bedside and be involved in decision-making […]' – please elaborate on the role played by enhancing patients' prognostic awareness (PA) by means of reiterated conversations on goals of care and shared decision-making (SDM) with clinicians which involve family members/caregivers as part of the holistic care dyadic to foster patients' (and caregivers') well-being and hope. Please expand on the role of involving multiple stakeholders and members in discussions around prognostication and how their involvement may enhance patients' PA and – ultimately – relieve their suffering and the related distress. To do so, please refer to and add citation of the following recent work: PMID: 39807018.

**Reviewer**	**2**
**Name**	**Siedner-Weintraub, Yael**
**Affiliation**	**Shaare Zedek Medical Center**
**Date**	**04-Sep-2025**
**COI**	**None**

I commend the authors on this study. This is an important issue examining a minority population, whose needs and wishes are not well described in the literature, and which many medical team members may not be familiar with, not just in the UK. The review of the papers found was done well and summarized succinctly, making it easy to read and understand, and to gain information for other medical providers.

Abstract -

There are some grammatical changes that should be made:

Line 7 - "ethnic diversities" - unclear. Please consider choosing a different term

Line 7 - "a third of whom is..." should be changed to "a third of whom are..."

Line 9-11 - Run-on sentence, please shorten or reword to improve clarity

Line 15 - "searched from 01-01-1994 and 07-10-2024" should be changed to "searched from 01-01-1994 to 07-10-2024"

Line 26 - "challenges to provide sensitive symptom..." should be changed to "challenges in provision of sensitive symptom..."

Introduction - I have no specific comments for the introduction.

Methods -

Literature search was done first by screening of titles, excluding more than 4000 studies. There is a chance that other studies, which may have had at least some reference to Muslims, were excluded in this way. This should be stated as a limitation of the study...

Please elaborate more on how you conducted the synthesis. In addition, only one author, JM, coded the initial studies, which opens potential biases. This should be stated in the limitations as well.

Results - In your methods, you describe a three-step plan for the study.

1. conducting a preliminary synthesis of findings; 2. exploring relationships between studies; 3. assessing the robustness of the synthesis.

The synthesis was done very clearly and make it very easy to understand. The results were described succinctly and in an organized fashion that helped in understanding different populations (patients, families, HCP) views' of Muslims at end-of-life.

Of note, in the table, some references have first and last name of first author, whereas some only state last name. It would help to have uniformity.

However, I'm not sure whether there was an exploration of the relationship between studies, were they in agreement? Did different studies highlight contradictory findings? (Sarah, Daniel - my understanding is that the "relationship" between the papers is whether they are in agreement. Am I right?). How did you assess the robustness of the synthesis?

Discussion - I would word the conclusions of this review more carefully. The Muslim faith community is large, and includes many sects (Shiite, Suni, etc.), and many cultural backgrounds (African communities, Middle East, South East Asia, etc.), which no doubt influence patients' needs, wishes and beliefs, specifically at end of life. Though the limitations are stated very clearly and thoughtfully (The lack of studies, the wide variety of Muslim traditions within the UK), the findings themselves are not worded to align with these very important limitations. This is particularly important for findings 1&2 - it is overstating the results to say that "conformity... is a priority for Muslim patients" (as in, all Muslim patients) and it is overstating the results to say that "Family-based care is... expected, to achieve a peaceful death".

It may be helpful to review this study: Abdulbaseer U, Piracha N, Hamouda M, Farajallah I, Abdul-Majid S, Abdelwahab S, Teltser WK, Padela AI. Muslim Patients' Religious & Spiritual Resource Needs in US Hospitals: Findings from a National Survey. J Gen Intern Med. 2025 Feb;40(2):376-384. doi: 10.1007/s11606-024-08960-y. Epub 2024 Sep 23. PMID: 39313670; PMCID: PMC11802954. into the discussion.

This study looks at a large number of Muslim patients in the USA, specifically at religious needs, and may help in underlining the studies still required ahead, as well as informing your discussion about needs of the Muslim population.

VERSION 1 - AUTHOR RESPONSE

Reviewer 1, Dr. Monaco’s comments:

Comment raised	Response by author	Location of revisions
In short, this is simply a fabulous investigation, which I have read with extreme interest. I am persuaded that the presented work adds to the existing body of knowledge in this field. The manuscript is genuinely wonderfully written, and no major linguistic and/or lexical inaccuracies are present. The Figure and Table add additional value to the authors' work. The methodology is strong and robust, the PRISMA checklist was followed in detail, prior registration on PROSPERO was executed, the search strategy and study selection process is described in detail, the results are presented objectively, and the discussion constitutes a well-balanced opportunity to elaborate more on the research findings.	We sincerely thank you for your time to review our article submission and your supportive comments.	N/A
I believe a comprehensive Figure in the form of a 'Visual/Graphical abstract' should be added to the manuscript which provides to provide a visual representation of the study background/aims, methodology, results and implication for clinical practice.	Thank you for this creative suggestion. After careful consideration, we have not done this as we need to abide by the submission guidelines of figures at BMJ Open. However, we have created a poster for this work as a graphical illustration of the abstract, which was presented at the Palliative Care Congress 2025. This can be viewed on the corresponding's author official LinkedIn page.	N/A

Please consider widening the scope of your ‘Introduction’ section by implementing some of the considerations which emerged from another recent article on this topic, i.e., PMID: 34860584.	Thank you for suggesting this article by Madni et.al which serves as an initiation point for further exploration of Muslim patients’ needs at the end of life. While widening the scope of the introduction allows for the consideration of the variance across different end of life care needs among Muslims, we chose to focus on symptom management needs in particular, as the aim of this review is to explore Muslims’ perspectives and preferences on end-of-life symptom management. Additionally, the methods of the suggested article do not allow for a primary, in-depth, and sensitive exploration of Muslims needs, and thus risks the overrepresentation of religious doctrines as opposed to patients’ individual perspectives.	N/A
In your ‘Discussion’ section, suggest also mentioning that tensions between religious and secular identities may impact spiritual care provision in the patient population of terminally ill patients, and that research focusing on the spiritual care needs of Muslims in Israel conducted by Baddarni et al. indicated that this tension may be less relevant for Israeli Muslims. Please refer to and add citation of both i) PMID: 39093919 and ii) Baddarni K. [Spiritual Care for the Muslim Patient] Spirit Care Muslim Patient Artic Hebr 2013; 26–36.	Thank you for your suggestions. The first article you referred to (PMID: 39093919) is a Bibliometric Network Analysis focused on spiritual care in palliative medicine; however, our review’s aim is to focus on symptom management within end-of-life care. Therefore, we are unfortunately unable to draw on findings from this study. The second article you suggested by Baddarni et al. is in written and published in Hebrew, and none of our study team speaks Hebrew. However, the point you raise about how different sects within the Muslim faith can affect end of life symptom management perspectives and preferences is a crucial point that remains under-explored in research. Consequently, we have added this as a limitation of our review findings and cited the following article: Suleman M. The balancing	Page 12

	of virtues—Muslim perspectives on palliative and end of life care: Empirical research analysing the perspectives of service users and providers. Bioethics. 2023 Jan;37(1):57–68	
In your 'Discussion' section, you state that: 'Dixon et al., (2021) identified extended families felt it was their duty to be present at the bedside and be involved in decision-making […]' – please elaborate on the role played by enhancing patients' prognostic awareness (PA) by means of reiterated conversations on goals of care and shared decision-making (SDM) with clinicians which involve family members/caregivers as part of the holistic care dyadic to foster patients' (and caregivers') well-being and hope. Please expand on the role of involving multiple stakeholders and members in discussions around prognostication and how their involvement may enhance patients' PA and – ultimately – relieve their suffering and the related distress. To do so, please refer to and add citation of the following recent work: PMID: 39807018.	Thank you for this suggestion. We appreciate that we did not specify what type of decision making that extended families felt responsible for sharing when reporting on the Dixon et al. study. As such, we provided more details in relation to this study. We also expanded on the role of involving multiple stakeholders in care discussions by referring to the following article:<br><br>Dionne-Odom JN, Ejem D, Wells R, Barnato AE, Taylor RA, Rocque GB, et al. How family caregivers of persons with advanced cancer assist with upstream healthcare decision-making: A qualitative study. Vaismoradi M, editor. PLOS ONE. 2019 Mar 13;14(3):e0212967.	Page 11

Reviewer 2, Dr. Siedner-Weintraub's comments:		
Comment raised	Response by author	Location of revisions
I commend the authors on this study. This is an important issue examining a minority population, whose needs and wishes are not well described in the literature, and which many medical team members may not be familiar with, not just in the UK. The review of the papers	We sincerely thank you for your time to review our paper and your supportive feedback.	

found was done well and summarized succinctly, making it easy to read and understand, and to gain information for other medical providers.		
There are some grammatical changes that should be made:<br>Line 7 - "ethnic diversities" - unclear. Please consider choosing a different term<br>Line 7 - "a third of whom is..." should be changed to "a third of whom are..."<br>Line 9-11 - Run-on sentence, please shorten or reword to improve clarity<br>Line 15 - "searched from 01-01-1994 and 07-10-2024" should be changed to "searched from 01-01-1994 to 07-10-2024"<br>Line 26 - "challenges to provide sensitive symptom..." should be changed to "challenges in provision of sensitive symptom..."	Thank you for your keen review. Suggested grammatical changes have been implemented to the abstract.	Page 2
Literature search was done first by screening of titles, excluding more than 4000 studies. There is a chance that other studies, which may have had at least some reference to Muslims, were excluded in this way. This should be stated as a limitation of the study...	Thank you for your comment. We applied a systematic approach to mitigate against this and do not consider it to be a limitation. We were aware that title screening had the potential to exclude articles with some reference to Muslims that was not included in the title. As such, all articles with titles that could not enable us to definitively rule out their relevance to our review questions were moved to the abstract screening phase – with two reviewers independently screening at each stage and an inclusive approach adopted. Similarly, all abstracts of which we were in doubt were moved to full-text screening to ensure that we did not inadvertently exclude any relevant articles.	N/A

Please elaborate more on how you conducted the synthesis. In addition, only one author, JM, coded the initial studies, which opens potential biases. This should be stated in the limitations as well.	Thank you for your suggestions. We have done this in the methods section as requested.<br><br>Potential biases related to having one author coding data and leading the thematic analysis have been lessened by the independent Weight of Evidence scoring by two members of the team, and reflexive, iterative discussions with authors BB and KS. A point in relation to this has also been added to the transcript in the methods. This limitation you raise has also been added to the discussion section as a separate paragraph	Pages 9 and 12
Of note, in the table, some references have first and last name of first author, whereas some only state last name. It would help to have uniformity.	Thank you for spotting this. We have now included only the last names of authors in Table 3, which is now provided as supplemental document 3 due to BMJ Open's criteria of the length and width of tables allowed in the manuscripts.	Supplemental document 3
I'm not sure whether there was an exploration of the relationship between studies, were they in agreement? Did different studies highlight contradictory findings?	Thank you for your questions. An exploration of the relationship was carried out as a component of the narrative synthesis and embedded throughout the results section. The themes identified are the overall homogenous findings of included studies. However, within each theme, we also highlight areas of heterogeneity across findings of included papers. We have made sure to highlight those instances throughout the results section.	Pages 9-10
How did you assess the robustness of the synthesis?	Thank you for your question. This was aided through the application of Gough's Weight of Evidence scores and through reflexive discussions with two other authors at key stages. The included studies were re-reviewed to check the robustness of final synthesis and	Pages 5-6

	ensure analytical themes were grounded in the primary data. Additional text has been added to further detail these steps.	
I would word the conclusions of this review more carefully. The Muslim faith community is large, and includes many sects (Shiite, Suni, etc.), and many cultural backgrounds (African communities, Middle East, South East Asia, etc.), which no doubt influence patients' needs, wishes and beliefs, specifically at end of life. Though the limitations are stated very clearly and thoughtfully (The lack of studies, the wide variety of Muslim traditions within the UK), the findings themselves are not worded to align with these very important limitations. This is particularly important for findings 1&2 - it is overstating the results to say that "conformity... is a priority for Muslim patients" (as in, all Muslim patients) and it is overstating the results to say that "Family-based care is... expected, to achieve a peaceful death".	Thank you for your suggestion. We agree. The wording of the conclusion and summary of findings in the discussion section has been improved and aligned with the limitations discussed. As recommended, points 1 and 2 of 'summary of findings' in the discussion section were rephrased.<br><br>We have highlighted the limitation of the existing evidence in the conclusion section too.	Pages 11 and 13
It may be helpful to review this study: Abdulbaseer U, Piracha N, Hamouda M, Farajallah I, Abdul-Majid S, Abdelwahab S, Teltser WK, Padela AI. Muslim Patients' Religious & Spiritual Resource Needs in US Hospitals: Findings from a National Survey. J Gen Intern Med. 2025 Feb;40(2):376-384. doi: 10.1007/s11606-024-08960-y. Epub 2024 Sep 23. PMID: 39313670; PMCID: PMC11802954. into the discussion. This study looks at a large number of Muslim patients in the USA, specifically at religious needs, and may help in underlining the studies still required ahead, as well as informing your discussion	Thank you for your suggestion. While this is an insightful study in its own right, it does not specifically relate to Muslims' end-of-life care experiences and therefore, we are unable to draw on its findings to directly support the points we raise in the first paragraph of our discussion section. However, we did draw on findings from the wider international literature on Muslim communities end-of-life care perspectives, such as those from: Klitzman R, Di Sapia Natarelli G, Garbuzova E, Sinnappan S, Al-Hashimi J. Muslim patients in the U.S. confronting challenges	N/A

about needs of the Muslim population.	regarding end-of-life and palliative care: the experiences and roles of hospital chaplains. BMC Palliat Care. 2023 Mar 27;22(1):28.	

VERSION 2 - REVIEW

Reviewer 1

Name Monaco, Fabrizio

Affiliation Scientific Institute San Raffaele, Cardiothoracic and Vascular Anesthesia

Date 21-Nov-2025

COI

I thank the Editor and the Authors for allowing me an opportunity to review the revised version of the manuscript ‘Muslim Communities' Perspectives and Preferences Regarding End-of-Life Symptom Management: A Systematic Review and Narrative Synthesis’ submitted for consideration of publication in the 'BMJ Open' Journal.

First off, I would like to thank the authors for their outstanding revision effort, which has been - in this Reviewer's opinion - really beneficial.

All of this Reviewer's comments have now been adequately addressed in this rebuttal, resulting in a much stronger and more in-depth analysis of this interesting topic, which will definitely add value to this Journal.

Taking this expert Reviewer's original requests for revision into account, the Authors' responses to my queries and an additional round of reading of the final version of the manuscript, I would now recommend for its acceptance.

Reviewer 2

Name Siedner-Weintraub, Yael

Affiliation Shaare Zedek Medical Center

Date 06-Dec-2025

COI

Wonderful job!
